# Fatal facial necrotizing fasciitis: a case report highlighting diagnostic and therapeutic challenges

**DOI:** 10.1093/omcr/omaf137

**Published:** 2025-08-20

**Authors:** Muhammad Sheraz Hameed, Syed Rafay Hussain Zaidi, Ali Iqbal, Muhammad Ahsan, Austin Mitchell, Azka Asad Mirza, Joshiah Gordon, Rahmat Gul Omarzai

**Affiliations:** Department of Medicine, Rawalpindi Medical University, Rawalpindi, Pakistan; Department of Medicine, Uc-Health Parkview Medical Center, Pubelo,USA; Department of Medicine, Thomas Jefferson University Hospital, Philadelphia, USA; Department of Medicine, Federal Medical and Dental College, Islamabad, Pakistan; Department of Medicine, Uc-Health Parkview Medical Center, Pubelo,USA; Department of Medicine, Al-Nafees Medical College (Isra University), Islambad, Pakistan; Department of Medicine, Uc-Health Parkview Medical Center, Pubelo,USA; Department of Medicine, Nangarhar Medical University, Jalalabad, Afghanistan

**Keywords:** Fasciitis, Necrotizing, Face, Soft Tissue Infections, Sepsis, Multiple Organ Failure

## Abstract

**Background:**

Facial Necrotizing Fasciitis (FNF) is a rare but aggressive, life-threatening infection involving the face's subcutaneous tissues and underlying musculature. It can rapidly progress to septic shock and multi-organ failure if not promptly recognized and treated. The clinical course, including severe pain, disproportionate tenderness, and systemic signs, is often the most crucial factor in diagnosis. While computed tomography (CT) can aid in identifying early signs such as soft tissue swelling and gas formation, clinical suspicion remains paramount and should not be delayed by reliance on imaging alone. Without early intervention, FNF may lead to devastating outcomes, including disfigurement, blindness, or death.

**Case Presentation:**

We present the case of a 70-year-old male who developed facial necrotizing fasciitis with rapid systemic progression. He initially presented to the emergency department with acute tenderness and swelling of the right ear. CT imaging revealed extensive edema and swelling in the subcutaneous fat of the face, scalp, and neck, most prominently involving the right external ear. Despite aggressive supportive measures—including ventilatory, cardiovascular, and renal support—the infection progressed to severe multi-organ failure, ultimately resulting in death.

**Conclusion:**

This case highlights the fulminant course and high mortality risk associated with FNF. Early recognition based on clinical evaluation, supported—but not replaced—by imaging, is essential for diagnosis. Prompt intervention with broad-spectrum antibiotics and surgical debridement offers the best chance to improve outcomes and prevent catastrophic complications such as septic shock, multi-organ failure, and death.

## Introduction

Necrotizing fasciitis (NF) is a rare but rapidly progressive, life-threatening subcutaneous infection most commonly attributed to Group A Streptococcus that affects the skin and subcutaneous tissue. It is characterized by widespread tissue necrosis that rapidly advances along the superficial layers, involving the subcutaneous cellular tissue, skin, and underlying muscles [1] This rare condition presents significant diagnostic and management challenges for clinicians and is associated with a high mortality rate. Although imaging modalities such as computed tomography (CT) and magnetic resonance imaging (MRI) are valuable in confirming the diagnosis and assessing the extent of the infection, the clinical course and presentation are the most critical factors in early recognition and intervention. NF typically develops in areas where the skin barrier is disrupted, such as after surgery or trauma, allowing microbial invasion. However, it can also develop in previously healthy tissue.

Alcoholism, diabetes mellitus, renal failure, malignancy, immunodeficiency, nutritional disorders such as obesity, increasing age, and even metabolic syndrome in young adults are common predisposing factors for NF [2, 3]. Prompt recognition is crucial, followed by early treatment with intravenous antibiotics and extensive surgical debridement [4, 5]. Facial necrotizing fasciitis is particularly challenging to diagnose due to the complexity of the facial anatomy and its overlap with other conditions, such as cellulitis. The condition typically presents with severe pain that is disproportionate to physical findings, along with erythema, edema, and elevated temperature [6]. The progression of symptoms, such as increasing pain, systemic signs (e.g., fever, tachycardia), and the development of systemic involvement (e.g., septic shock, multi-organ failure), should raise suspicion for NF, especially in high-risk patients. Its rapid progression and systemic involvement—such as septic shock, reduced level of consciousness, and multi-organ failure—make early recognition critical [5, 6].

Mortality with necrotizing fasciitis (NF) is high due to systemic involvement. There are four types of necrotizing fasciitisNF: type I is the most prevalent (70–80% of cases) and is polymicrobial; type II is due to monomicrobial infection with Group A Streptococcus alone; type III is due to infection with Clostridium species or gram-negative bacteria, and type IV is due to fungal infections [7, 8].

Facial involvement is a rare form of necrotizing fasciitisNF, with a reported incidence in the United Kingdom of 0.24 per 1,000,000 per year [2]. Due to the high vascularity of this region, morbidity and mortality associated with facial NF are rare because of high antibiotic penetration [3]. Systemic involvement in this form is even more uncommon. However, here we present a case of facial necrotizing fasciitisNF (FNF) that progressed to septic shock and death.

## Case presentation

**Figure 1:**
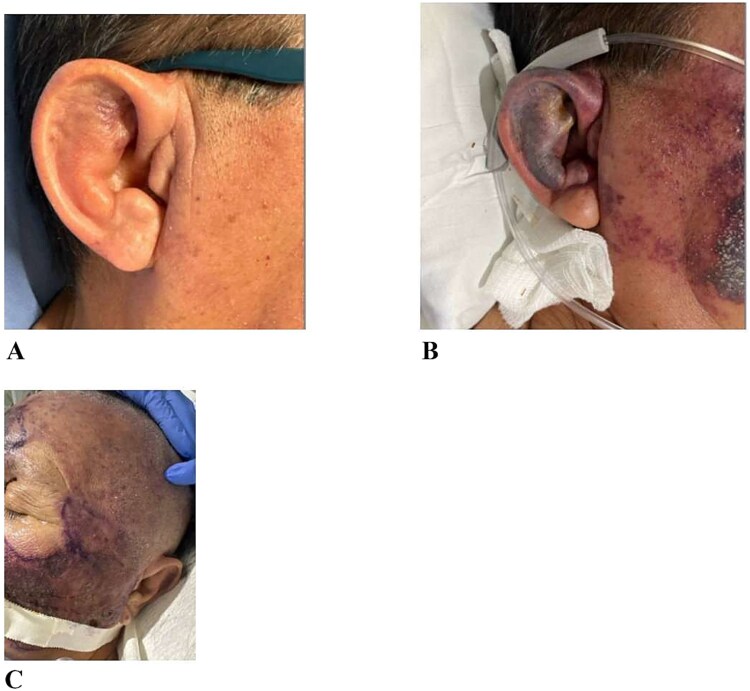
(**A**) Clinical appearance at the time of presentation, showing no external signs of facial or scalp swelling. (**B**) Image taken six hours after presentation, showing early progression of swelling in the facial and scalp regions. (**C**) Diffuse swelling of the face and scalp noted 12 hours after presentation, indicating rapid progression of soft tissue edema.

A 70-year-old male with a past history of type 2 diabetes mellitus, smoking, uncontrolled hypertension, hyperlipidemia, benign prostatic hyperplasia, prostate cancer, and gastroesophageal reflux disease presented to the emergency department with dizziness, weakness, and severe, excruciating pain and swelling in his face and right ear (Figure 1). There was no history of previous trauma, sinus disease, or recent surgery. He also felt extremely dizzy upon ambulation. The patient reported having 4–5 loose bowel movements, which had since resolved. However, within 12 hours, the patient developed diffuse facial swelling.

**Table 1:** HM-23 CBC-Diff profile

**Test**	**Results**	**Units**	**Reference range**
WBC Total	6.5	**/**μl	(4000/μl – 11000/μl)
RBC Total	4.22	m/μl	M (4.5-6.5) m/μl F (3.8-5.8) m/μl
Hemoglobin	12.8	g/dl	M (13.0-18.0) g/dl F (11.6-16.5) g/dl
HCT	41	%	M (40-50) % F (38-47) %
MCV	97.2	fL	(80-90) fL
MCH	30.3	pg	(27-32) pg
MCHC	31.2	g/dl	(33-38) g/dl
Platelet Count	134000	/ul	(150,000-400,000)/μl
Neutrophils	93.8	%	(40-75) %
Lymphocytes	7	%	(20-45) %

On examination, his blood pressure was 84/56 mmHg, with a heart rate of 104 beats per minute, a temperature of 99.7°F, a respiratory rate of 23/min, and SpO₂ of 96%. Tenderness, swelling of the right ear, and mastoid tenderness were noted. The mucous membranes of the mouth were dry. Abdominal tenderness was present in the right upper quadrant, with a positive Murphy sign. Capillary refill was also delayed.

**Figure 2:**
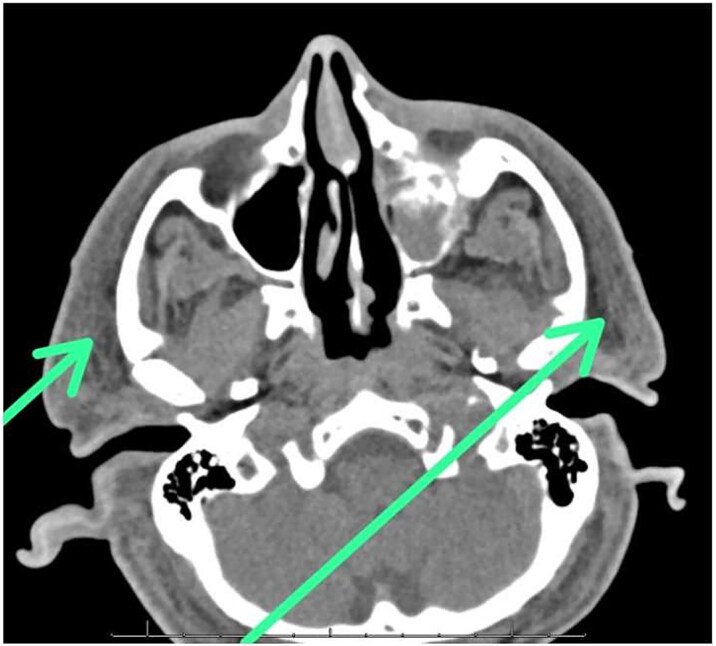
Axial CT image showing subcutaneous fat swelling in the face, most prominently involving the right external ear. Asymmetrical soft tissue thickening is also noted along the right ear region.

Laboratory tests revealed an increased procalcitonin level (20.27), suggesting an inflammatory response. Serum creatinine (2.80) and blood urea nitrogen (35) were elevated, indicating acute kidney injury (Table 2). Additional findings included lactic acidosis, with a lactic acid level of 6.0. Liver function tests were also abnormal (Table 3). Blood culture was positive for Group A Streptococcus (Table 4). Abdominal ultrasound showed cholecystitis. The CT scan of the face showed extensive edema and swelling in the subcutaneous fat of the face, scalp, and neck, particularly involving the right external ear. Increased asymmetrical soft tissue thickening was also noted along the right ear (Figure 2).

**Table 2:** Hematology

**Tests**	**Results**	**Units**	**Reference range**
Lactate Serum	6.0	mmol/L	(0.5-2.2)mmol/L
Procalcitonin	20.27	μg/L	(0.0-0.05) μg/L
Creatinine	2.80	mg/dl	(0.6-1.2) mg/dl
Blood Urea Nitrogen	35	mg/dl	(5-20) mg/dl
Blood Glucose	10.3	mmol/L	(4.0-5.6) mmol/L

**Table 3:** LFTs

**Tests**	**Results**	**Units**	**Normal value**
Alkaline phosphatase	202	IU/L	(30-140) IU/L
Alanine aminotransferase	82	IU/L	(7-56) IU/L
Aspartate aminotransferase	72	IU/L	(8-33) IU/L
Bilirubin Total	1.0	mg/dl	(0.2-1.3) mg/dl
Bilirubin Direct	0.5	mg/dl	(0.0- 0.2)

**Table 4:** Blood Culture

**Culture site**	**Organism**	**Sensitivity**
Blood	Streptococcus A pyogenes	Ampicillin, Vancomycin, Gentamicin and linezolid

The diagnosis of facial necrotizing fasciitis in this case was made using clinical criteria, supported by imaging studies. Initially, the patient presented with significant pain, erythema, and swelling of the facial tissues, with no external injury. We utilized the Laboratory Risk Indicator for Necrotizing Fasciitis score to assess risk, but imaging studies were the primary determinant in confirming the diagnosis. MRI was used to identify fascial involvement, and its high sensitivity provided clear evidence of tissue necrosis.

Antimicrobial Therapy: Broad-spectrum antibiotics were started immediately upon consultation with the infectious disease team. The following regimens were initiated; Piperacillin/tazobactam (4.5 g every 6 hours intravenously) for polymicrobial coverage, Vancomycin (1 g every 12 hours intravenously) for methicillin-resistant Staphylococcus aureus coverage, Clindamycin (600 mg every 8 hours intravenously) for Group A Streptococcus and toxin inhibition, Metronidazole (500 mg every 8 hours intravenously) for anaerobic coverage. Apart from patient was on IVIG, levophed (norepinephrine) and intravenous fluid for toxic shock.

No surgical intervention or transfer to a specialized facility was performed. Given the rapid progression of the disease and the patient’s critical condition, a decision was made to continue medical management with close monitoring.

Despite this treatment, the patient continued to deteriorate rapidly. Within a few hours of the presentation, he developed respiratory compromise and hemodynamic instability, necessitating intubation for ventilatory support. The progressive decline in blood pressure raised concerns about ongoing septic shock. The patient subsequently developed acute kidney and liver failure. Polymicrobial NF was suspected, and an emergency transfer to a specialized center for surgical intervention was planned. However, his condition continued to worsen, and he died from multi-organ failure and septic shock within 36 hours

## Discussion

Necrotizing fasciitis of the face is a rare subtype of head and neck infections. The hallmark of this condition is rapidly spreading soft tissue necrosis, which can result in severe deformity and potentially fatal outcomes [4–6]. It is characterized by systemic toxicity, arterial thrombosis, and extensive fascial necrosis. Polymicrobial pathogens—including Streptococcus pyogenes, Staphylococcus aureus, and Bacteroides spp.—can invade fascial planes through traumatic breaches in skin or mucosa, or via odontogenic infections [5, 7]. These microorganisms accelerate tissue destruction and evade immune defenses by producing exotoxins and proteolytic enzymes [5]. The face’s rich vascular supply and venous drainage facilitate rapid disease progression to the orbits, cranial base, and mediastinum, increasing the risk of multi-organ failure, cavernous sinus thrombosis, and airway obstruction [5, 7]. Host factors such as trauma, poor oral hygiene, and malnutrition further increase susceptibility to infection [7].

Necrotizing fasciitis (NF) of the face is a rapidly progressive infection with distinct symptoms and high morbidity. Early diagnosis is often difficult, as severe, disproportionate pain at the infection site usually precedes visible skin changes . The condition can progress within hours from erythema and swelling to dark discoloration and extensive necrosis [5, 9]. Systemic toxicity—manifesting as vomiting, confusion, and high fever (>102°F)—indicates sepsis and multi-organ involvement [6]. In reported cases, sepsis typically develops within 24–48 hours, and despite aggressive treatment, mortality rates can exceed 25% [5].

Facial NF is frequently misdiagnosed as cellulitis or angioedema, which delays appropriate treatment [9–11]. As many as 85% of early cases lack classical signs such as crepitus, hemorrhagic bullae, or systemic toxicity, leading to delayed surgical intervention [11, 12]. Odontogenic causes may present with dysphagia or trismus, further obscuring the diagnosis, especially when classic signs like "dishwater pus" or visible necrosis are absent [11]. Although the Laboratory Risk Indicator for Necrotizing Fasciitis (LRINEC) score can assist in risk stratification, it has limited sensitivity (59%) and reduced accuracy in patients with renal disease [13, 14]. Imaging techniques such as MRI (100% sensitivity) and CT (94% specificity for fascial fluid) support the diagnosis but should not delay surgical exploration [15, 16]. Despite potential false negatives in early disease, intraoperative confirmation via blunt dissection ("finger test") and histopathological analysis remains the gold standard [12].

Due to the complex anatomy of the face, rapid progression of disease, and high mortality risk, management of facial NF requires a multidisciplinary approach. Plastic surgeons for reconstruction, infectious disease specialists for targeted antibiotic therapy, and ENT or maxillofacial surgeons for airway protection and infection source control all play essential roles [17, 18]. Because multi-organ failure occurs in 30–50% of cases, critical care specialists are vital in managing sepsis [16]. Early surgical debridement is crucial to preserving facial nerves and vital structures, and serial debridements should be performed every 48–72 hours. Reconstructive surgery should be delayed for 3–7 days after infection control to enhance tissue viability [17]. Although facial NF has a lower mortality rate (12–28%) than truncal NF, its potential complications—such as mediastinitis and cavernous sinus thrombosis—necessitate thoracic surgery consultation in about 15% of cases [18].

In our case, a combination of systemic complications, rapid disease progression, and delayed surgical intervention led to a fatal outcome. The diagnosis was made clinically, and within 12 hours of presentation, the infection had spread rapidly despite prompt medical management, resulting in diffuse facial and scalp swelling. By the time the patient was transferred for ENT evaluation and surgical debridement, systemic toxicity, and extensive tissue necrosis had already developed. The face’s rich vascular and lymphatic network facilitated rapid microbial dissemination, increasing the risk of sepsis, multi-organ failure, and cardiovascular collapse. Despite aggressive supportive measures, the patient died within 36 hours of presentation.

Long-term complications of NF, especially in patients with underlying comorbidities such as diabetes mellitus, renal impairment, or immunosuppression, may include chronic pain, functional disability due to extensive tissue loss, disfigurement, and psychological trauma. Survivors often require prolonged rehabilitation and multiple reconstructive surgeries. Studies have shown that patients with underlying conditions are at higher risk of poor wound healing and recurrent infections, underscoring the need for multidisciplinary follow-up and individualized care plans post-discharge

Although facial necrotizing fasciitis can be aggressive, deaths directly due to sepsis and multi-organ failure are exceedingly rare [5]. However, several factors influence patient outcomes. Significant risk factors include advanced age and comorbidities such as coronary artery disease, diabetes mellitus, and renal impairment [18]. Prompt diagnosis and early initiation of broad-spectrum antibiotics are essential for favorable outcomes [5, 20]. Surgical debridement remains the cornerstone of infection control, but facial involvement presents distinct challenges due to aesthetic and functional concerns [5]. A careful balance must be maintained between preventing disfigurement and performing necessary aggressive surgical interventions. Additionally, comprehensive intensive care is crucial to managing sepsis and minimizing the risk of multi-organ failure [19, 20]. Optimizing outcomes in facial NF requires a multifactorial strategy incorporating early diagnosis, timely but judicious surgery, effective antibiotic therapy, and robust critical care support.

Several studies have demonstrated the critical importance of early intervention in improving outcomes in cases of FNF. Misiakos EP et al. reported that early recognition and intervention are pivotal for reducing morbidity and mortality in FNF cases [21]. Similarly, Nawijn F et al. highlighted that surgical debridement within the first 24 hours of diagnosis significantly reduces mortality [22]. Goh T et al. and Matalon et al. further emphasized the need for prompt diagnosis and treatment, suggesting that delayed diagnosis and treatment often leads to systemic involvement and worse outcomes [23, 24].

This case underscores a potential missed opportunity for earlier diagnosis and intervention. The delay in recognizing the severity of soft tissue involvement, compounded by the subtle early signs of facial necrotizing fasciitis, may have contributed to the progression to septic shock. Earlier imaging, surgical consultation, and broader-spectrum antibiotics could potentially have altered the clinical course. This highlights the need for a high index of suspicion and aggressive management, even when classical signs are not prominent in atypical locations such as the face.

## Conclusion

Facial necrotizing fasciitis is a rare but severe, life-threatening condition marked by rapid progression to septic shock and multi-organ failure, often resulting in fatal outcomes. Early recognition through vigilant clinical assessment and timely imaging—particularly CT—is critical for initiating prompt intervention. Immediate initiation of broad-spectrum intravenous antibiotics, combined with urgent surgical debridement, remains the cornerstone of effective management. These measures are essential to limit disease spread, reduce the risk of systemic complications, and improve survival outcomes.

## Consent to publish

Patient agreed to publish material. Informed Consent was Obtained from the patient.

## Guarantor

Muhammad Sheraz Hameed

muhammadsherazhameed786@gmail.com

Rawalpindi medical university, Rawalpindi, Pakistan.

## Data Availability

Data will be provided on request by corresponding author
